# GL-Segnet: Global-Local representation learning net for medical image segmentation

**DOI:** 10.3389/fnins.2023.1153356

**Published:** 2023-04-03

**Authors:** Di Gai, Jiqian Zhang, Yusong Xiao, Weidong Min, Hui Chen, Qi Wang, Pengxiang Su, Zheng Huang

**Affiliations:** ^1^School of Mathematics and Computer Sciences, Nanchang University, Nanchang, China; ^2^Jiangxi Key Laboratory of Smart City, Nanchang, China; ^3^Institute of Metaverse, Nanchang University, Nanchang, China; ^4^School of Software, Nanchang University, Nanchang, China; ^5^Office of Administration, Jiangxi Provincial Institute of Cultural Relics and Archaeology, Nanchang, China

**Keywords:** neuroscience, medical image segmentation, vision transformer, Global-Local representation learning, multi-scale feature fusion

## Abstract

Medical image segmentation has long been a compelling and fundamental problem in the realm of neuroscience. This is an extremely challenging task due to the intensely interfering irrelevant background information to segment the target. State-of-the-art methods fail to consider simultaneously addressing both long-range and short-range dependencies, and commonly emphasize the semantic information characterization capability while ignoring the geometric detail information implied in the shallow feature maps resulting in the dropping of crucial features. To tackle the above problem, we propose a Global-Local representation learning net for medical image segmentation, namely GL-Segnet. In the Feature encoder, we utilize the Multi-Scale Convolution (MSC) and Multi-Scale Pooling (MSP) modules to encode the global semantic representation information at the shallow level of the network, and multi-scale feature fusion operations are applied to enrich local geometric detail information in a cross-level manner. Beyond that, we adopt a global semantic feature extraction module to perform filtering of irrelevant background information. In Attention-enhancing Decoder, we use the Attention-based feature decoding module to refine the multi-scale fused feature information, which provides effective cues for attention decoding. We exploit the structural similarity between images and the edge gradient information to propose a hybrid loss to improve the segmentation accuracy of the model. Extensive experiments on medical image segmentation from Glas, ISIC, Brain Tumors and SIIM-ACR demonstrated that our GL-Segnet is superior to existing state-of-art methods in subjective visual performance and objective evaluation.

## 1. Introduction

Medical image segmentation is one of the vital technologies for intelligent analysis and understanding of medical images, providing clinicians with significant information for diagnosis and treatment (Ali et al., 2020). Specifically, in the planning of radiotherapy, it can accurately depict the area where the tumor is located to maximize the coverage of the target area. Tumor delineation is usually performed manually or semi-manually, which requires highly specialized knowledge, and is time-consuming for the annotator (Sutton et al., 2020). Therefore, image segmentation of lesion areas through deep learning to assist doctors has been the focus of research for many years (Li et al., 2014; Mortazi and Bagci, 2018). Currently, medical image segmentation has been applied to multiple organs, such as liver segmentation (Li et al., 2015; Vorontsov et al., 2018), brain tumor segmentation (Cherukuri et al., 2018), cell segmentation (Li et al., 2018), heart segmentation (Khened et al., 2018), etc. The traditional method of extracting features is normally designed using expert knowledge according to the gray value, shape, and texture to automatically segment the target region. For instance, threshold segmentation method (Oksuz et al., 2022), cluster segmentation method (Hua et al., 2021), region-based segmentation method (Xiao-yao et al., 2020) and edge detection-based segmentation method (San-ping et al., 2017). The above methods frequently involve a large amount of prior knowledge to extract manual features for segmentation. Nevertheless, the designed manual features are only valid for segmentation tasks on specific datasets and the segmentation performance is not stable. The segmentation method based on deep learning adopts the idea of pixel classification, which is different from the traditional method of manually extracting features. Deep learning-based methods have flourished in the field of medical image segmentation (Gai et al., 2020; Liu et al., 2021; Touvron et al., 2021). Li et al. (2020) introduced encoder and decoder structures into the field of image segmentation and proposed the Fully Convolutional Network (FCN), which preserves the location information by replacing the fully connected with convolutional layers. U-Net (Ronneberger et al., 2015) used contraction paths to obtain feature information and expansion paths to achieve precise positioning, which has preeminent performance on various data sets. Chu and et al. (2020) proposed a method that utilizes a simple edge detector to locate all discontinuities and additionally monitor these regions, which effectively improves the segmentation accuracy. Although the model based on the convolutional neural network has excellent representation ability, it is difficult to model the features with long-range dependencies in the image because the convolutional computation has only a fixed receptive field, failing to capture sufficient contextual information.

The main contributions of this work are as follows:

1. To encode the global semantic representation information at the shallow level of the network, we employ the MSC and MSP modules. Meanwhile, the Multi-scale feature fusion operation was adopted by us to cross-level enrich the local geometric detail information.
2. We utilize an attention-based feature decoding module to generalize the feature information, which provides effective clues for attention decoding.
3. We exploit the structural similarity between images and the edge gradient information to propose a hybrid loss, which protects image edge information and improves the performance of the model.
4. The proposed model achieves excellent segmentation results on GLAS, ISIC, Brian Tumors and SIIM-ACR datasets.

## 2. Related work

### 2.1. Medical image segmentation

Medical image segmentation aims to make the human tissue or pathological structures vibrant and intuitive. In addition, the relevant tissue can be modeled through the segmentation results for subsequent manipulation. Early, image segmentation methods were mainly divided into threshold-based segmentation methods (Tang et al., 2017), region-based segmentation methods (Deng et al., 2019), edge-based segmentation methods (Borovec et al., 2017), and segmentation methods based on specific theories (Liu et al., 2022). At present, SegNet (Badrinarayanan et al., 2017) directly extracted target features and achieved unexceptionable segmentation performance. Kitrungrotsakul et al. (2020) proposed an interactive deep optimization network for medical image segmentation. In addition, Gu et al. introduced a CE-Net model for medical image segmentation, which involves a dilated convolution to change the receptive domain size of the model and reduce information loss. An Edge Attention Network is proposed, which embedded edge attention representations to guide the segmentation network (Zhijie et al., 2019). Although CNN networks have a great advantage in the extraction of local features, they are less capable of encoding global information.

### 2.2. U-shaped network structure

A U-shaped network structure based on FCN is widely used in medical image segmentation. U-Net is applied in numerous fields of segmentation, which has an outstanding contribution in the medical and biological fields (Liu et al., 2020b). The role of the encoder is to accomplish feature extraction, which can be done using various classical convolutional neural networks such as VGG, Inception, ResNet, DenseNet, etc. All these networks can be used as the encoding layer, while in the decoding layer, the opposite operation can be performed. On this basis, Shankaranarayana et al. (2017) combined the idea of residual connectivity with U-Net to propose the residual U-Net. Oktay et al. (2021) exploited the Attention U-Net to capture salient features by integrating attention gates. Zongwei et al. (2020) proposed U-Net++, which fixes features at different levels and utilizes a flexible network structure with deep supervision, enabling deep networks to drastically reduce the number of parameters within acceptable accuracy. Apart from that, Jafari et al. (2020) added additional skip connections to Residual Network (ResNet) (Kaiming et al., 2016) and Dense Convolutional Network (DenseNet) (Huang and Wang, 2017) to reduce the time complexity (Song et al., 2018). The CNN network has great advantages in the extraction of local features, but it lacks the ability to encode contextual information.

### 2.3. Transformer mechanism

Compared to Convolutional Neural Network (CNN), Transformer (Vaswani et al., 2017) effectively establishes long-range dependencies through the Self-Attention mechanism. Zheng et al. (2021) applied the Transformer as an encoder to compress the spatial resolution, progressively extracting high-level semantic features and mapping the features to the original spatial resolution through a decoder for final pixel-level segmentation. Petit et al. (2021) proposed the U-Transformer network structure, which develops Multi-Head Self-Attention to obtain remote dependencies, resulting in the excellent recovery of spatial resolution. Zhang et al. (2021) fused two parallel CNN branches and Transformer branches to attain global dependencies and local detail features, using AGs to fuse multi-level features between different layers. Moreover, Valanarasu et al. (2021) employed a Local-Global training strategy to extract geometric features such as details and textures through shallow global branching. The deep local branching is involved to extract spatial location information to obtain the final segmentation result. Since the Transformer cannot capture the internal relationship between each slice, Chu et al. (2021) added a conditional position encoding generator to produce an implicit position encoding that allows the original spatial position relationship. Recently, Chen et al. (2021) proposed Transununet by exploiting the advantages of Transformer and U-Net. In the encoder part, the Transformer is presented to encode the feature map from the CNN, to enrich the contextual features. The encoded features are up-sampled in the decoder part to acquire precise localization. Although these methods achieved good results, they lack local area information interaction in the process of encoding (Liu, 2020a).

## 3. Method

### 3.1. Feature encoder

The proposed encoder is composed of two parts: a Context-rich connection module and a Global semantic feature extraction module. Among them, we focus on the various feature information of global and local contained in different layers using a variety of modules in context-rich operations, so as to solve the problem of detail loss during the upsampling process. In the Global semantic feature extraction module, The Residual-Block can tackle the problem that the gradient of the network disappears during the training process, thus improving the performance of the network; The Vision Transformer (ViT) module introduces a multi-headed attention mechanism into the network, which allows the network to reduce the interference of non-semantic feature information during the coding process.

**Figure 1:**
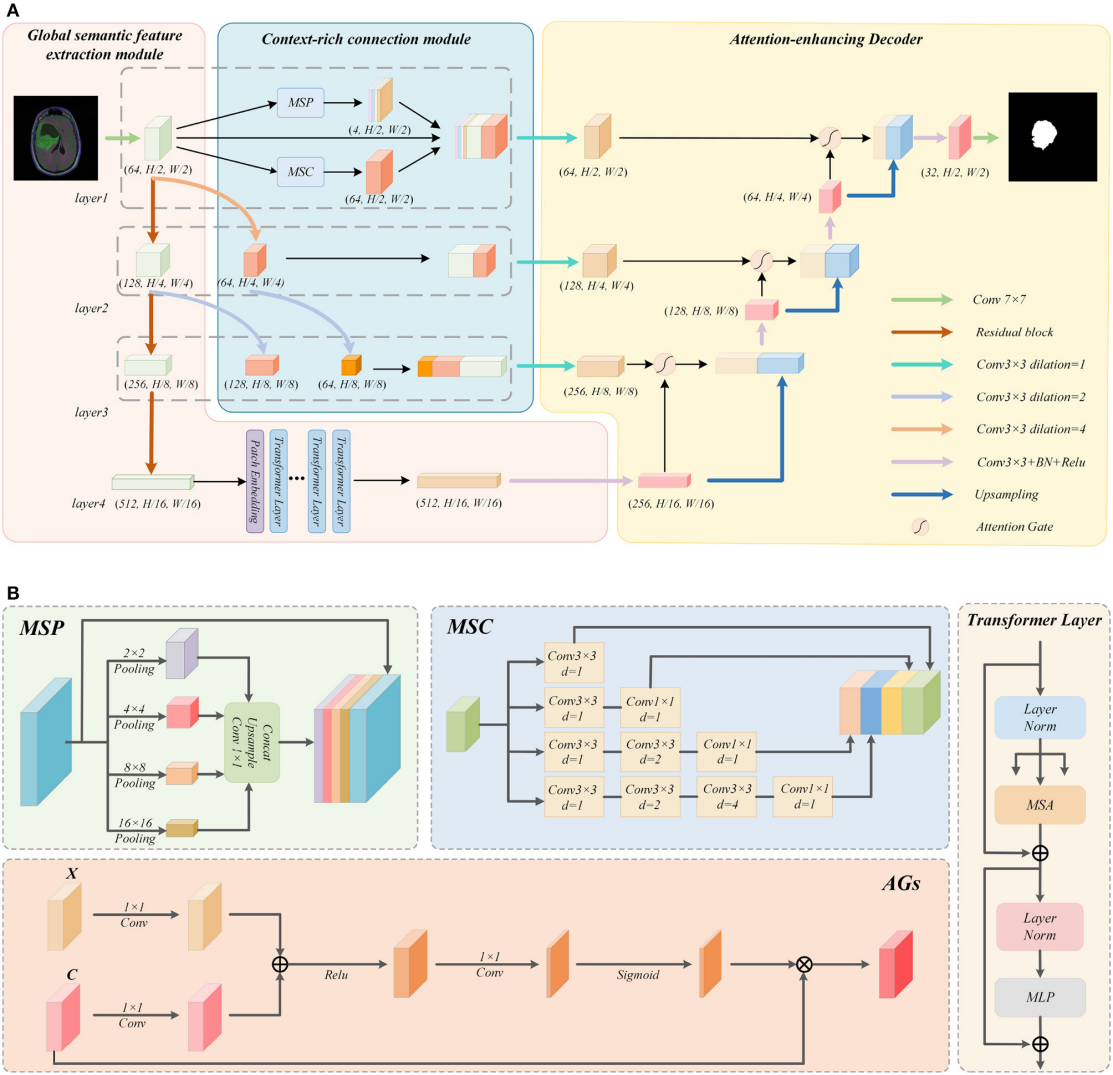
Detailed explanation of the GL-Segnet. **(A)** Overview of the GL-Segnet. **(B)** Illustrations of the modules.

As shown in Figure 1A, the Multi-Scale Convolution (MSC) and Multi-Scale Pooling (MSP) modules are used to perform a multi-scale fusion from the features in layer 1. Meanwhile, for the Context-rich operations of layer 2 and layer 3, we choose to perform atrous convolution operations with different atrous rates on the output of the upper Residual-block. After adjusting, it is concatenated with the output of this Residual-block layer as a feature supplement, so as to enrich the context of the network.

Concretely, the proposed encoder structure consists of four layers. First, a convolution operation is used to perform preliminary feature extraction on the input image. After each layer of the network has been passed down through the Residual-block, the size of the feature is changed to 1/2 of the original size and the number of channels is doubled. In the last layer, a high-level feature is established. Finally, the output of the encoder is processed by the multi-headed self-attention mechanism in the ViT module.

#### 3.1.1. Context-rich connection module

To encode the global semantic representation information at the shallow level of the network and cross-level enrich the local geometric detail information, a variety of modules are utilized to augment the semantic features of the context in the network. For the large-size and lower-level feature maps initially extracted by a simple convolution operation in layer 1, we apply multi-scale semantic feature enrichment operations using *MSC* and *MSP* modules. This operation can balance the geometric detail information representation capability and the semantic information representation capability of the feature map. Additionally, for the output of the other two layers of Residual-Block, we propose a multi-level feature fusion module. It uses atrous convolution for obtaining feature maps with different representation capabilities by changing the size of the receptive field. Finally, they are concatenated as skip connections to balance the global semantic representation information and local geometric detail information. Therefore, it is effective to cope with the feature loss phenomenon during upsampling.

**MSC Module:** As shown in Figure 1B, four different convolutions are used to process the input feature map, so as to obtain four features of the same size from multiple scales. Mathematically, the atrous convolution is computed as follows:


(1) $$\begin{array}{l} y\left[i\right]=\underset{l}{\sum }x\left[i+dl\right]w\left[l\right] \end{array}$$

Where *y*[*i*] is a point at position *i* of the output feature map, *x* is the input feature map, *w* is the convolution weight, *w*[*l*] is the point at position *l* of the convolution weight matrix, *l* denotes a coordinate pair representing position similar to (-1,1), and *d* is the atrous rate.

The four feature maps obtained in this way will contain different receptive fields and convolution depths. After the four feature maps are cascaded, the number of channels will be changed to 1/4 of the original feature map through a 3 × 3 convolution. Thus, these features are integrated to get a feature map with the same size and channel as the input.

Since atrous convolution has a grid effect, it cannot perform element-by-element calculations for a matrix. When operating on lower-level feature maps, the grid effect of atrous convolution may lead to the harm of detailed information.

**MSP Module:** As the pooling operation can compensate for the grid effect of the atrous convolution, we tend to use the MSP module to make up for this defect. Specifically, three different scale pooling operations are applied to obtain feature maps of three sizes. Afterward, they are turned into three channels of 1 and the same size as the input through a 1 × 1 convolution and upsampling operation. At the same time, when pooling is utilized to expand the receptive field, part of the semantic representation information extracted by the network will be lost, while atrous convolution can protect the extracted semantic representation information. The *MSP* module is described as follows:


(2) $$Y=\left[\begin{array}{c} UP(\mathrm{Conv}\left({\text{ Pooling }}_{2\times 2}(X)\right)\\ UP(\mathrm{Conv}\left({\text{ Pooling }}_{4\times 4}(X)\right)\\ UP(\mathrm{Conv}\left({\text{ Pooling }}_{8\times 8}(X)\right)\\ UP(\mathrm{Conv}\left({\text{ Pooling }}_{16\times 16}(X)\right) \end{array}\right]$$

Where *X* is the input, *Y* is the output, *UP* is the upsampling, *Conv* is the convolution with a kernel size of 1 and a channel number of 1, *Pooling*_*a*×*a*_ is the maximum pooling of size *a*, and [·] is the concatenate operation.

The feature map of size 64 × (*H*/2) × (*W*/2) is obtained by *MSC* module, and the feature map of size 4 × (*H*/2) × (*W*/2) is attained by *MSP* module, which is concatenated with the feature map extracted from Layer 1.

**Multi-scale feature fusion operation:** The Residual-blocks in Layer 2 and Layer 3 output feature maps with smaller sizes and high-level features. They are not suitable to use the *MSC* and *MSP* modules for feature enrichment operations as in Layer 1. To enrich the feature information of the local geometric detail of them that can be used in decoding, we proposed the multi-scale feature fusion operation. This operation first convolves the feature maps with different sizes of receptive fields, and then concatenates the feature maps of the same size after convolution. This will compensate and enrich the detailed features of the semantic feature information of the underlying network abstraction. Also reduce the loss of semantic features during simple upsampling.

In details, we firstly perform two consecutive atrous convolution operations on the feature maps in Layer 1 after the initial feature extraction with different atrous rates. By controlling the padding value, a feature map of size (*H*/4) × (*W*/4) and a feature map of size (*H*/8) × (*W*/8) can be obtained. Likewise, we also use the atrous convolution to operate on the feature map output by the Residual-Block in Layer 2 to obtain a feature map of size (*H*/8) × (*W*/8) by controlling the padding.

Then the feature maps of size (*H*/4) × (*W*/4) are concatenated with the feature maps output by Residual-block in Layer 2, while the feature maps of size (*H*/8) × (*W*/8) are concatenated with the feature maps output by Residual-block in Layer 3. These concatenated feature maps will be utilized in the skip connection to enrich the contextual information and thus reduce the loss of feature information during upsampling.

#### 3.1.2. Global semantic feature extraction module

In the structure of the proposed model, we combine Residual-block and ViT to create a novel encoder module. The encoder of the traditional U-Net adopts a convolution stacking structure, which fails to deal with the problem of gradient disappearance during the training process. Correspondingly, adding residual connections (Kaiming et al., 2016) can boost the performance of the network. Specifically, the formula for Residual connection is as follows:


(3) $$\begin{array}{l} Y=F\left(X,W_{i}\right)+X \end{array}$$

Where *X* is input, the *Y* is output, *F* is the convolution layer operation, and *W*_*i*_ is the convolution parameter.

As the background information is extracted into the feature map at the same time as the semantic information, it can cause the network to conduct non-semantic information during the decoding process, thus affecting the accuracy of the segmentation task. To decrease the interference of background information on semantic information, after extracting feature information from multiple stacked Residual-blocks, we employ the ViT module to filter the extracted features to obtain semantic feature information. The ViT module consists of Patch embedding and several Transformer layers.

The ViT module first uses a multi-layer Residual-block to accomplish feature extraction, which can obtain the feature map *H*×*W*×*C*. The feature map is then divided into N sub-blocks of size *P*×*P*×*C*, whose number is $\frac{\left(H\times W\times C\right)}{\left(P\times P\times C\right)}$. Then, they are stitched together horizontally to obtain a combined feature map of size *N*×(*P*×*P*×*C*). The combination of feature maps are compressed into a feature map *N*×*K* by performing a linear transformation through the fully connected layer. In addition, ViT introduces a trainable location embedding feature map to improve the location information. The Patch embedding specific formula is as follows:


(4) $$\begin{array}{l} M_{0}=\left[Y_{p}^{1}B;Y_{p}^{2}B;Y_{p}^{3}B;\cdots \;;Y_{p}^{N}B\right]+B_{pos} \end{array}$$

Where *B* is the fully connected layer of the embedding, and *B*∈*R*^(*P*×*P*×*C*) × *K*^, *B*_*pos*_ is the positional embedding feature map, and$B_{pos}\in R^{\left(N\times K\right)}$.

The Transformer layer mainly includes two parts, MSA and MLP. The output of the Lth layer is expressed by the following formulas:


(5) $$\begin{array}{l} \begin{array}{c} m_{l}^{\prime }=MSA\left(LN\left(m_{l-1}\right)\right)+m_{l-1}\\ m_{l}=MLP\left(LN\left(m_{l}^{\prime }\right)\right)+m_{l}^{\prime } \end{array} \end{array}$$

Where *LN*(·) is the normalization operator, *MSA* is the Multihead Self-Attention module, and *MLP* is the Multi-layer Perceptron module. The multi-head self-attention used in the *MSA* module is different from the common self-attention mechanism, which transforms the input feature map into three different matrices, namely, query matrix *Q*, key value matrix *K*, and value matrix *V*. The query matrix *Q* is multiplied by the transpose of the key value matrix *K* to obtain the similarity matrix *QK*^*T*^. The similarity matrix is normalized by the softmax function to obtain the weight matrix. The weight matrix is multiplied by the value matrix to get the attention of the input matrix, the specific formula is defined as:


(6) $$\begin{array}{l} \text{Attention}\left(\text{Q},\text{K},\text{V}\right)=\mathrm{softmax}\left(\frac{\text{Q}{\text{K}}^{\text{T}}}{\sqrt{d_{k}}}\right)\times \text{V} \end{array}$$

Where *d*_*k*_ represents the dimension of the query matrix or key value matrix. The *MSA* module is the core component of Transformer layer. It uses a multi-head attention mechanism, which is composed of n self-attention mechanism modules. The specific formula is:


(7) $$\begin{array}{l} \begin{array}{c} MSA\left(Q,K,V\right)=\left[h_{1};\ldots ;h_{n}\right]W^{O}\\ \text{where }{\text{h}}_{i}=\text{Attention}\left(QW_{i}^{Q},KW_{i}^{K},VW_{i}^{V}\right) \end{array} \end{array}$$

Where ${W_{i}}^{Q}$, ${W_{i}}^{K}$, ${W_{i}}^{V}$are the linear transformation matrices of the *i*−*th* self-attention mechanism. The *n* self-attention mechanism modules are concatenated and then multiplied with the linear transformation matrix *W*^*O*^ to obtain the final output.

The *MLP* is mainly composed of two fully connected layers and a linear activation layer *ReLU* linearly combined.


(8) $$\begin{array}{l} MLP\left(X\right)=\max\left(0,XW_{1}+b_{1}\right)W_{2}+b_{2} \end{array}$$

Where *W*_1_, *b*_1_ and *W*_2_, *b*_2_ represent the weights and biases of the two fully connected layers, respectively.

### 3.2. Attention-enhancing decoder

The Attention-enhancing decoder used in the proposed model consists of attention-based feature decoding module and cascaded upsampling module. The attention-based feature decoding module can make optimal use of the feature information in the skip connection feature maps. Moreover, it extracts useful semantic feature information from the redundant feature information in feature maps; The cascaded upsampling module enables the network to cope well with the loss of important semantic feature information during the upsampling process.

As shown in Figure 1A, for each layer of the decoder, we initially perform feature decoding on the feature maps which have been subjected to feature enrichment operations. In the process of feature decoding, the attention-based feature decoding module needs to use the output feature map of the upper layer network for assistance. Eventually, the feature maps after feature decoding are upsampled with the feature maps output by the upper layer network using the cascaded upsampling module. The final output image of the network, will be obtained by a 7 × 7 convolution operation after going through the decoder.

#### 3.2.1. Attention-based feature decoding module

In the decoding process, as the skip connection feature maps of each layer undergo semantic feature enrichment operations, they contain features extracted from several different scales and are richer in feature information. Nevertheless, overly redundant feature information may affect the performance of the decoding module. Therefore, a 3 × 3 convolution operation is used to perform a feature summarization operation on the skip connection feature map before adding the attention mechanism to it. After this, we use the feature maps output by the upper layer network for assistance, performing Attention-Gate operation on the feature maps after feature summarization.

**Attention-gate:** The application of Attention-Gate introduces the attention mechanism to the decoder, thus highlighting the semantic feature information conveyed by the feature maps after feature summarization, while reducing the interference of irrelevant background information to the semantic information during decoding. The structure of Attention-Gate is shown in Figure 1B. Where *X* is the feature map output from the upper layer of the network, the *C* is the skip connection feature map after the feature summarization operation mentioned above. Initially, perform a 1 × 1 convolution operation on them, and then sum the obtained outputs, which highlights the feature information contained in the two feature maps simultaneously. After the *Relu* function, it goes through a 1 × 1 convolution to make the feature map channel equals to 1. After normalizing the feature map by the *Sigmoid* function, it is multiplied with *C* to obtain the output of Attention-Gate. The specific formula is as follows:


(9) $$\begin{array}{l} AG\left(X,C\right)=\sigma_{2}\left(\psi \left(\sigma_{1}\left(W_{x}X+W_{c}C\right)\right)\right)C \end{array}$$

Where *X* and *C* have been mentioned above, *W*_*x*_ is the weight of the 1 × 1 convolution on *X*, *W*_*c*_ is the weight of the 1 × 1 convolution on *C*, σ_1_ is the *Relu* function, σ_2_ is the *Sigmoid* function, and ψ is the weight of the 1 × 1 convolution on the feature map after summation.

The soft attention operation of the multilayer Attention-Gate can effectively reduce the interference of the background feature information to the semantic information in the skip connection feature map, so as to obtain a segmented image with more accurate segmentation.

#### 3.2.2. Cascaded upsampling module

The module formula is as follows:


(10) $$\begin{array}{l} Y={\mathrm{Conv}}_{3\times 3}\left(\left[AG\left(X,C\right);UP\left(X\right)\right]\right) \end{array}$$

Where *X* is the feature map output from the upper layer network, *C* is the skip connection feature map mentioned above, *UP*(·) operation is the upsampling operation with twice magnification scale, *AG*(·) operation is the Attention-Gate operation described above, *Conv*_3 × 3_ is the convolution operation with 3 × 3 convolution kernel size, while [;]is the concatenate operation.

The upsampling module completes the decoding process from high-level features to segmentation masks. It is a combination of multiple upsampling steps, consistent with U-Net. By upsampling the high-level features and concatenating them with the skip connection feature maps before the convolution layer operation. This prevents the loss of some detailed features caused by mere upsampling operation during the image recovery process, thereby ensuring the accuracy of the recovered image.

### 3.3. Hybrid loss

The proposed model employs a hybrid loss function with multiple loss functions interacting with each other in order to balance the evenly decline of each metric during learning. The loss functions adopted include: Dice loss, Binary cross entorpy loss, SSIM loss and Edge preservasion loss. we assume that Dice loss is *L*_1_, Binary cross entorpy loss is *L*_2_, SSIM loss is *L*_3_, and Edge preservasion loss based on the gradient-based Edge preservasion loss is *L*_4_.

**Dice loss:** The Dice loss function is a common loss function applied in the field of image segmentation to measure the similarity of two sets, and its specific formula is:


(11) $$L_{1}=\frac{2\sum_{i}t_{i}e_{i}}{\sum_{i}t_{i}+\sum_{i}e_{i}}$$

Where *i* denotes a pixel point, *t*_*i*_ is whether the current pixel point is the semantic pixel point in the ground truth, and *e*_*i*_ is whether the current pixel point is classified as a semantic pixel point in the predicted image.

Dice loss can reflect the image similarity well from the region, and has good performance for the scenario with serious imbalance between positive and negative samples, so we choose it as the main loss function of Hybrid loss.

**Binary cross entropy loss:** The binary cross entropy loss function is a common loss function for binary classification problems, which is a convex optimization function. It facilitates us to find the optimal value by gradient descent method, while being able to measure the subtle differences between the two pictures. The specific formula of this loss function is as follows:


(12) $$L_{2}=\frac{-\left(\sum_{i}\left(t_{i}*\log\left(e_{i}\right)+\left(1-t_{i}\right)*\log\left(1-e_{i}\right)\right)\right)}{N}$$

where *N* denotes the total number of pixel points. The meaning of *i*, *t*_*i*_, *e*_*i*_ in the formula is the same as in Dice loss.

**SSIM loss:** SSIM loss is used to measure the structural similarity between two images. It measures the similarity between two images by brightness, contrast, and structure. The addition of SSIM loss enables us to obtain higher quality images. the formula for calculating *SSIM* is as follows:


(13) $$\begin{array}{l} \mathrm{SSIM}\left(I_{1},I_{2}|\omega \right)=\frac{\left(2{\bar{\omega }}_{1}{\bar{\omega }}_{2}+C_{1}\right)+\left(2\sigma_{\omega_{1}\omega_{2}}+C_{2}\right)}{\left({\bar{\omega }}_{1}^{2}+{\bar{\omega }}_{2}^{2}+C_{1}\right)\left(\sigma_{\omega_{1}}^{2}+\sigma_{\omega_{2}}^{2}+C_{2}\right)} \end{array}$$

Where ω_1_ and ω_2_ are the chunked images of *I*_1_ and *I*_2_ respectively, ${\bar{\omega }}_{1}$ and ${\bar{\omega }}_{2}$ are the mean values of ω_1_, ω_2_ images respectively, σ_ω_1_ω_*n*__ is the covariance of ω_1_ and ω_2_ of the two images. σ_ω_1__ and σ_ω_2__ are the variance of ω_1_ and ω_2_ respectively. The larger *SSIM* value of the two pictures, the greater the structural similarity of the two pictures, so when *SSIM* is used as a loss function, we take:


(14) $$\begin{array}{l} L_{3}=1-\mathrm{SSIM}\left(I_{s},I_{g}\right) \end{array}$$

where *SSIM*(*I*_*s*_, *I*_*g*_) denotes the average of *SSIM* of all windows of both *I*_*s*_ and *I*_*g*_ images.

**Edge preservasion loss:** In semantic segmentation, the edge information of the semantic region is most likely to be lost during encoding and decoding, thus, we introduce a gradient-based edge-preserving loss function, the expression of which is:


(15) $$L_{4}=\frac{1}{H\times W}\left\|\Delta I_{S}-\Delta I_{g}\right\|$$

where Δ*I*_*s*_ and Δ*I*_*g*_ are the gradients of *I*_*s*_ and *I*_*g*_, which are calculated as follows (using Δ*I*_*s*_ as an example)


(16) $$\begin{array}{l} \begin{array}{c} \Delta I_{s}=\frac{\partial^{2}I_{s}\left(x,y\right)}{\partial x^{2}}+\frac{\partial^{2}I_{s}\left(x,y\right)}{\partial y^{2}}\\ \frac{\partial^{2}I_{s}\left(x,y\right)}{\partial x^{2}}=I_{f}\left(x+1,y\right)+I_{f}\left(x-1,y\right)-2I_{f}\left(x,y\right)\\ \frac{\partial^{2}I_{s}\left(x,y\right)}{\partial y^{2}}=I_{f}\left(x,y+1\right)+I_{f}\left(x,y-1\right)-2I_{f}\left(x,y\right) \end{array} \end{array}$$

The same formula for Δ*I*_*g*_ can be easily obtained.

In this paper, we utilize a combination of the above four loss functions to form the hybrid loss function *L*.

The expressions are:


(17) $$\begin{array}{l} \begin{array}{c} L=\alpha L_{1}+\beta L_{2}+\gamma L_{3}+\theta L_{4}\\ \text{where }\alpha +\beta +\gamma +\theta =1 \end{array} \end{array}$$

## 4. Experiment

### 4.1. Experimental datasets

**GLAS (Glad segmentation) dataset:** a public dataset from the MICCAI 2015 challenge, consisting of 165 images from 16 H&E (hematoxylin and eosin) stained slides of colorectal cancer tissue sections. The original images varied in size, mostly 775 × 522. To facilitate training, we preprocessed the dataset into images of size 256 × 256. The dataset was separated into training set and test set, in which 144 images were divided into training set and 36 images were divided into test set.

**ISIC2018 dataset:** This dataset is a dataset for skin lesion analysis for melanoma detection, in which the part for medical image segmentation includes 5460 RGB skin lesion images. The dataset was divided into training set and test set. There were 3461 images of training set and 2002 images of test set.

**Brain tumors dataset:** This dataset was chosen from those mentioned in the paper (Mazurowski et al., 2017; Buda et al., 2019), which were obtained from The Cancer Imaging Archive TCIA and The Cancer Genome Atlas. It includes brain slice images of 110 LGG patients, and after processing the dataset, we obtained a total of 1311 brain images of various sizes. We divided the dataset into two parts: the training set and the test set, including 1049 images as training set and 262 images as test set.

**SIIM-ACR dataset:** This dataset consists of partial anteroposterior chest radiographs from the public dataset of the pneumothorax X-ray segmentation and recognition competition held by the Society for Imaging Informatics in Medicine in August 2019, with 101 X-ray chest images. (101 labeled data in nii format, with 2 being the lungs, 3 being the heart, and 0 being the background in the labeled data.) We performed correlation processing on this dataset, retaining the lung labels from the labeled data and using the lung images as the segmentation target for these experiments. We divided the dataset into a training set and a test set, with 81 images as the training set and 20 images as the test set.

### 4.2. Implementation details

The proposed network was implemented based on the PyTorch architecture, and an NVIDIA TITAN V GPU was used to accelerate our experiments. During the experiments, we resized all the datasets into 256 × 256 pixel images and chose the SGD optimizer with momentum to train the network, where the momentum size was 0.9, the learning rate was 0.01, and the weight decay parameter in the optimizer was 0.0001. For experiments on GLAS and SIIM-ACR datasets, we performed 400 iterations. For the Brian Tumors dataset, we performed 100 iterations; for the ISIC dataset, we performed 30 iterations.

### 4.3. Evaluation indicators

In order to evaluate the performance of the model more comprehensively and accurately, five evaluation indices are chosen to evaluate the results of our experiments in various aspects, including Dice Coefficient (DICE), Intersection over Union (IoU), Weighted F-measure (wFm), Enhanced-alignment Metric (Em), and Structure-based Metric (Sm). These evaluation indices reflect the degree of strengths and weaknesses of different aspects of the model. Among them, the Dice and IoU indices are used to evaluate the similarity degree between the pixel points of two image collections; the wFm index intuitively generalizes the F-measure by calculating the accuracy and recall rates alternately. It extends the four basic quantities Tp, Tn, Fp, and Fn to real values and considers the neighborhood information to give different weights to different errors at different positions, thus highlighting the target part of the evaluation by weighting. The Em index can reflect both image-level statistical information and local pixel matching information between two image collections; the Sm index is a harmonic indicator of two structural similarity indicators, region-oriented and object-oriented, which can effectively respond to the structural similarity between two image collections.

### 4.4. Experimental results and analysis

To verify the accuracy of our model and reflect the segmentation effect of our model, we selected eight state-of-the-art CNN-based networks for comparison, including U-Net (Ronneberger et al., 2015), Segnet (Badrinarayanan et al., 2017), R2U-Net (Alom et al., 2018), Attention U-Net (Oktay et al., 2021), R2AU-Net (Zuo et al., 2021), BiSeNet-V2 (Yu et al., 2021), KiU-Net (Valanarasu et al., 2020), and Transunet (Chen et al., 2021). In the following, we will introduce the specific situation of the experiment based on each of the four datasets mentioned above.

#### 4.4.1. GLAS dataset

**Figure 2:**
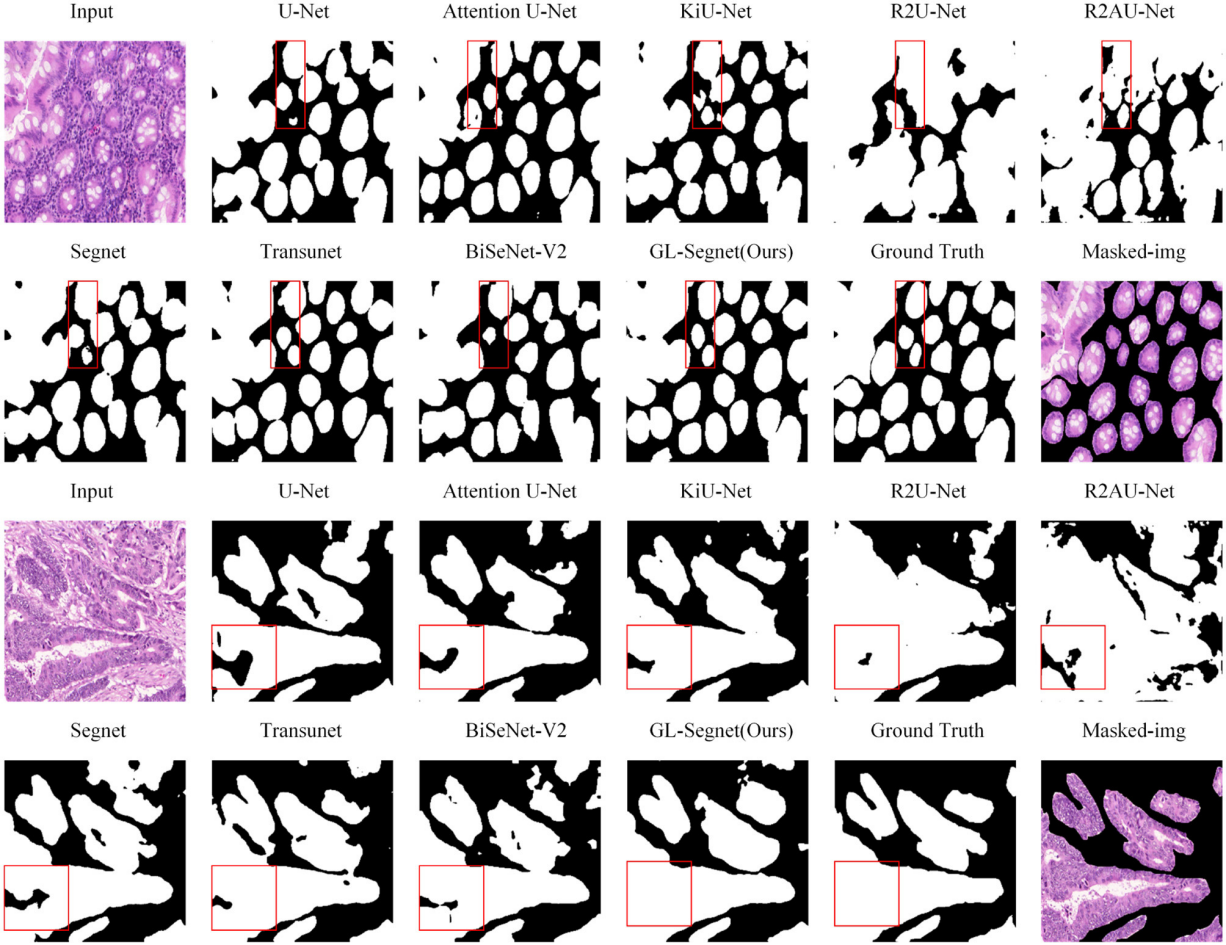
Qualitative results on GLAS dataset.

**Figure 3:**
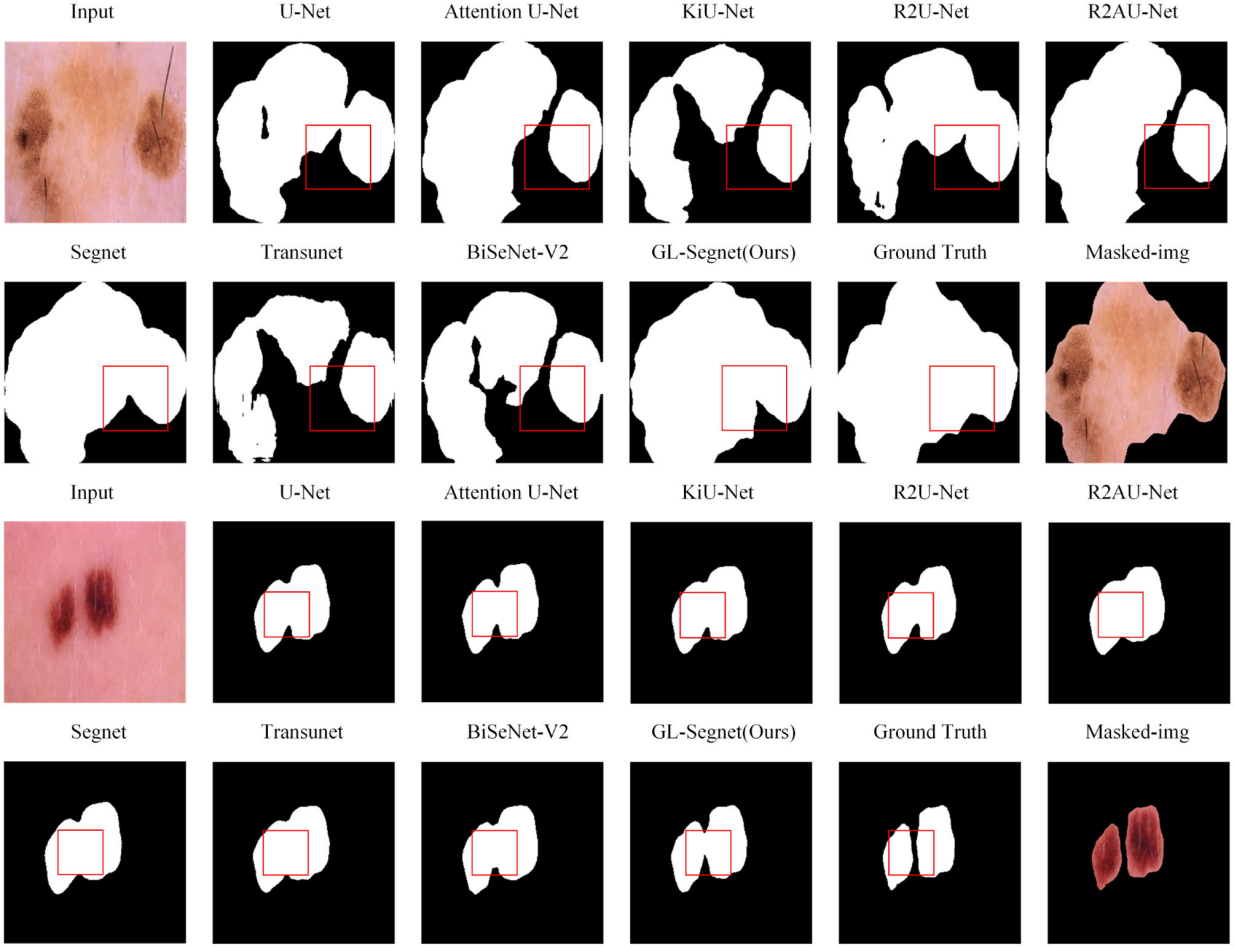
Qualitative results on ISIC dataset.

Figure 2 shows the visual comparison results between the other models and the proposed model on the GLAS dataset. As shown by the segmentation effect of the red rectangular box labeled area in Figure 2, our network performs better, with segmentation results closest to the Ground Truth and fewer under-segmented tissue regions. In the first set of comparison experiments shown in Figure 3, the networks U-Net, KiU-Net, R2AU-Net and Segnet showed significant under-segmentation when segmenting the target glandular cells at the labeled region due to the absence of an attention mechanism. Specially, the BiSeNet-V2 network did not identify the target glandular cells. Adoption of the attention mechanism allows the network to reduce the interference of background information and hence segment the semantic targets more accurately, so the under-segmentation of target regions is enhenced in the prediction results of Attention U-Net and R2AU-Net compared to U-Net and R2U-Net. Our model and the Transunet model introduce a multi-head attention mechanism to further improve the network's resistance to extraneous background information, which makes a more accurate segmentation of the target glandular cells at the lower right corner of this annotated region. However, for the upper right glandular cells in this region, the Transunet model showed blurred boundaries, which were noticeably improved by introducing Edge preservasion loss to protect the edge information during the training of our model. Furthermore, in the second set of comparison experiments, only our model does not show any missing regions, benefiting from the usage of the multi-head attention mechanism, compared to the other models.

As shown in Table 1, overall, our method slightly outperformed the other methods in all indices on the GLAS dataset, with MeanDice and MeanIoU reaching 0.931 and 0.874, respectively. It can be seen that the overall effect of the proposed model is better. At the same time, our model is 1% and 0.8% higher than the two suboptimal methods in wFm and Sm, respectively, which is much higher than the level. In the Em index, our model also achieves the optimal value.

**Table 1:** The Quantitative result on the Glas dataset.

**Dataset**	**Method**	**MeanDice**	**MeanIoU**	**wFm**	**Sm**	**Em**
Glas	U-Net (Ronneberger et al., 2015)	0.915	0.847	0.902	0.845	0.911
	Segnet (Badrinarayanan et al., 2017)	0.925	0.863	0.913	0.854	0.919
	R2U-Net (Alom et al., 2018)	0.858	0.784	0.842	0.786	0.853
	Attention U-Net (Oktay et al., 2021)	0.929	0.870	0.920	0.860	0.922
	R2AU-Net (Zuo et al., 2021)	0.915	0.850	0.910	0.837	0.900
	BiSeNet-V2 (Yu et al., 2021)	0.921	0.856	0.912	0.847	0.916
	KiU-Net (Valanarasu et al., 2020)	0.923	0.860	0.922	0.854	0.918
	Transunet (Chen et al., 2021)	0.928	0.868	0.924	0.859	0.924
	GL-Segnet (Ours)	**0.931**	**0.873**	**0.930**	**0.868**	**0.928**

The highest value is marked bold.

Meanwhile, compared with U-Net, Segnet, and R2U-Net without the attention mechanism, our network is 1.7%, 0.9%, and 7.5% higher in Em parameter, respectively. This reflects that with the addition of the attention mechanism, the network can better reduce the interference of background information on semantic information.

#### 4.4.2. ISIC dataset

**Figure 4:**
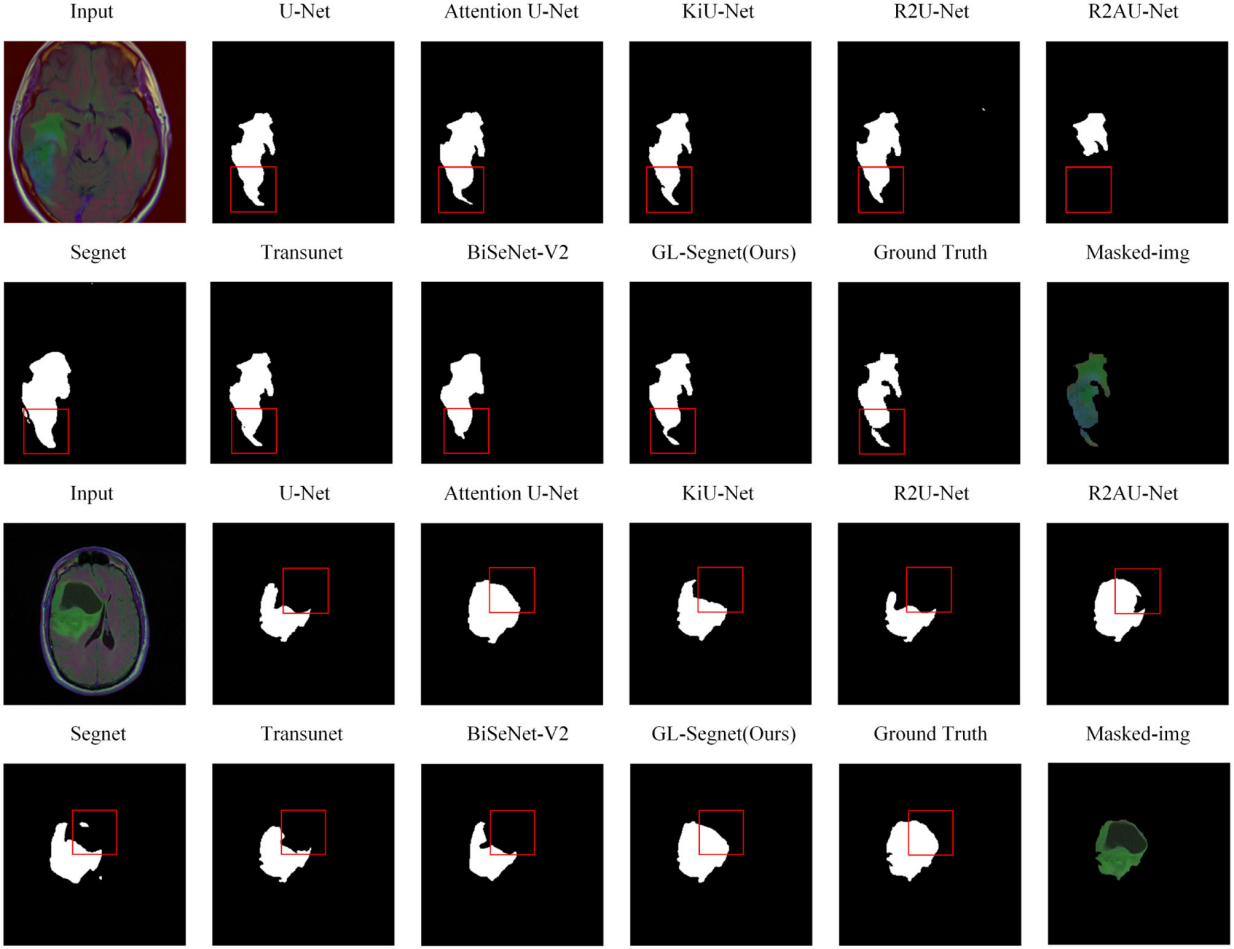
Qualitative results on Brain tumors dataset.

Figure 3 shows the visual comparison results of other models and the proposed model on the ISIC dataset. As the example images in columns 1 and 2 in Fig. 4, U-Net, Attention U-Net, KiU-Net, R2U-Net, R2AU-Net, Transunet, and BiSeNet-V2 networks all have obvious under-segmentation phenomena, while Segnet and the model proposed in this paper do not have obvious under-segmentation. However, the Segnet model suffers from an obvious under-segmentation of the boundary detail information. Meanwhile, in the area marked by the box in Figure 3, other networks failed to fully capture the edge details of the segmented lesions, and the unclear edge boundaries were not well handled. The proposed model used the Residual-Block module, improving the ability of the network to learn features. Hence, the proposed model can capture the edge details of the lesion, better handle the segmentation details, and achieve more accurate segmentation for the case of uneven grayscale and unclear edges in the segmented image.

As shown in Table 2, on the ISIC dataset, the proposed model is optimal in every evaluation index. Compared to the eight models, Segnet and Transunet have lower segmentation accuracy on the ISIC dataset, with Dice index of around 88%. U-Net, R2U-Net, AttentionU-Net, BiSeNet-V2, and KiU-Net are closer in accuracy, with the Dice index around 90%, and the rest of the indices are very close. R2AU-Net performs better than them in accuracy, while the proposed model is comparable to R2AU-Net in terms of accuracy, with a Dice index only 0.01% higher than it. In other indices, the proposed model is all slightly better than the second-best R2AU-Net model. GL-Segnet outperforms BiseNet-V2 and Transunet by 1.1 and 3.2 percentage points, respectively, in the Sm evaluation metric. This indicates that the proposed Attention-enhancing decoder of this network has better results in the extraction of semantic information compared with the more recent popular transformer.

**Table 2:** The Quantitative result on the ISIC dataset.

**Dataset**	**Method**	**MeanDice**	**MeanIoU**	**wFm**	**Sm**	**Em**
ISIC	U-Net (Ronneberger et al., 2015)	0.950	0.844	0.890	0.891	0.932
	Segnet (Badrinarayanan et al., 2017)	0.875	0.798	0.842	0.858	0.904
	R2U-Net (Alom et al., 2018)	0.907	0.845	0.898	0.893	0.933
	Attention U-Net (Oktay et al., 2021)	0.907	0.847	0.890	0.892	0.932
	R2AU-Net (Zuo et al., 2021)	0.913	0.853	0.902	0.896	0.938
	BiSeNet-V2 (Yu et al., 2021)	0.904	0.840	0.89	0.889	0.932
	KiU-Net (Valanarasu et al., 2020)	0.894	0.826	0.875	0.879	0.924
	Transunet (Chen et al., 2021)	0.880	0.807	0.859	0.868	0.913
	GL-Segnet (Ours)	**0.915**	**0.858**	**0.906**	**0.900**	**0.940**

The highest value is marked bold.

#### 4.4.3. Brian Tumors dataset

Figure 4 displays visual comparisons of the proposed model's comparison experiment using the Brain Tumors dataset. The proposed model introduces context-rich operations such as *MSC* and *MSP* modules, which enrich the features that the network can use, and the use of Residual-Block also allows the network to acquire more detailed information in the image. As a result, the network model can better deal with segmentation details. For example, in the box markers of the images in the first set of comparison experiments in Figure 4, except for the R2AU-Net model, which has a phenomenon of missing targets at the markers, the segmentation effect of the rest of the networks is good. However, the segmentation effect of the proposed model is significantly better than the other models for the detailed part of the “crabfoot-like” changes of the glial brain tumor at the box markers. Meanwhile, compared with the proposed network, the Transunet model only takes into account the long-distance dependence of images and the fusion of contextual information, but not the local dependence of images and the importance of channel information in the process of fusion of contextual information, so its target is missing in the necrotic area of the glioma marked by the box in the second set of comparison experiments in Figure 4. In comparison, Attention U-Net, R2AU-Net, and the network proposed introduce the Attention Gate structure in the process of decoding, which makes the network take into account the importance of channel information in the process of fusion of image and contextual information, so no target missing phenomenon occurs. The use of *MSC* and *MSP* modules in the proposed model allows the network to take into account the long-range dependence of images and the local dependence of images, making the proposed model more effective than AttentionU-Net and R2AU-Net and closer to the results of manual segmentation by doctors.

As shown in Table 3, on the TCGA dataset, the proposed model outperformed other methods in all indices. Compared to U-Net, Segnet, R2U-Net, Attention U-Net, R2AU-Net, BiSeNet-V2, KiU-Net, and Transunet, our model improved on MeanDice by 0.9%, 10.0%, 4.2%, 2.4%, 8.9%, 1.6%, 1.0%, and 0.5%, respectively. And it improved by 2.7%, 15.5%, 6.0%, 3.9%, 10.7%, 2.8%, 1.8%, and 0.8%, respectively, on the MeanIoU. The proposed model also outperformed the suboptimal model by 0.9%, 0.05%, and 0.02% on wFm, Sm, and meanEm, respectively, so it is evident that the *MSC* and *MSP* modules can extract multi-dimensional information, which enables subsequent attention mechanisms to make better use of multi-level feature information.

**Table 3:** The Quantitative result on the Brain Tumors dataset.

**Dataset**	**Method**	**MeanDice**	**MeanIoU**	**wFm**	**Sm**	**Em**
Brian Tumors	U-Net (Ronneberger et al., 2015)	0.922	0.861	0.925	0.937	0.983
	Segnet (Badrinarayanan et al., 2017)	0.831	0.722	0.790	0.858	0.928
	R2U-Net (Alom et al., 2018)	0.889	0.817	0.884	0.917	0.965
	Attention U-Net (Oktay et al., 2021)	0.907	0.836	0.892	0.922	0.975
	R2AU-Net (Zuo et al., 2021)	0.842	0.770	0.845	0.889	0.925
	BiSeNet-V2 (Yu et al., 2021)	0.915	0.849	0.920	0.932	0.983
	KiU-Net (Valanarasu et al., 2020)	0.921	0.859	0.927	0.937	0.985
	Transunet (Chen et al., 2021)	0.926	0.869	0.928	0.940	0.986
	GL-Segnet (Ours)	**0.931**	**0.877**	**0.937**	**0.945**	**0.988**

The highest value is marked bold.

#### 4.4.4. SIIM-ACR dataset

**Figure 5:**
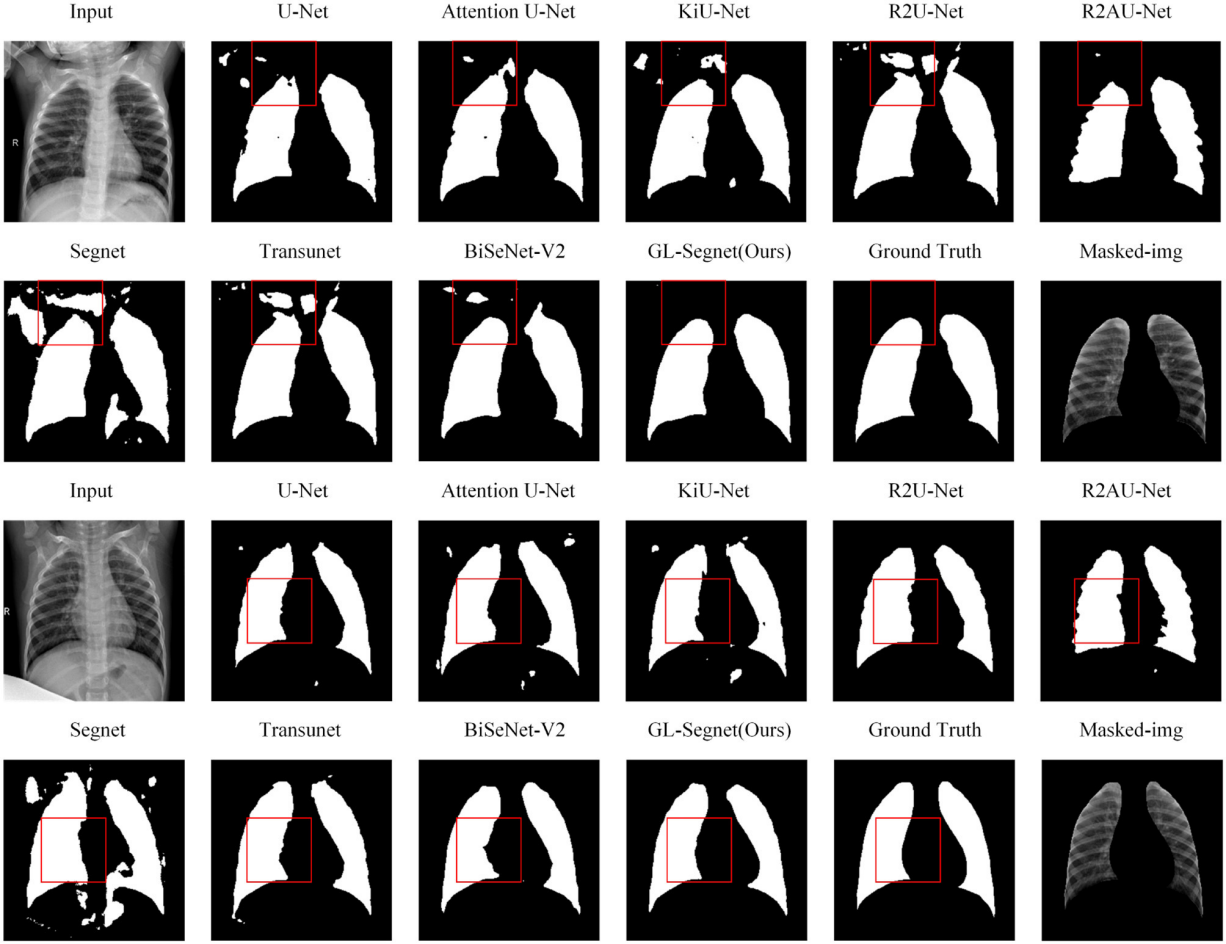
Qualitative results on SIIM-ACR dataset.

Figure 5 shows the visual comparison results between state-of-the-art models and propose model on the SIIM-ACR dataset. The region-based lung segmentation method is simple in calculation and fast in segmentation, but it is parameter-sensitive and cannot accurately segment the inter-adhesive lung regions. The introduction of the multi-headed attention mechanism and Residual-Block enables the proposed model to extract semantic feature information more accurately while reducing the interference of background feature information on the segmentation task, thus enabling the network to segment the inter-adherent lung regions more accurately. As shown in the comparison results of the box-labeled regions in the first set of comparison experiments in Figure 5. Except for the proposed model and Attention U-Net, other networks have obvious deviations when segmenting targets, including a lot of Mis-segmented regions, and cannot guarantee the integrity of segmentation results. At the same time, as the proposed network introduces the loss function of edge information protection in the training process, the model can segment the edge information of the target more accurately, as shown in the box-labeled regions of the images in the second set of comparison experiments in Figure 5. The lung lobe edges of the prediction results of other comparison experiments, except the proposed model, show obvious jaggedness. As for the predictions of the Attention U-Net model, which does not show obvious redundant regions in the prediction results of the images in columns 1 and 2, its segmentation results of the lung lobe edges also show obvious jaggedness. However, the predicted image edges of the propose model are smooth and are closest to the Ground truth manually labeled by the doctor.

As shown in Table 4, the proposed model improved MeanDice, wFm, and Sm indices by 0.09% over the suboptimal model on the SIIM-ACR dataset, and its MeanIoU and meanEm indices increased significantly compared to Transunet, KiU-Net, and Attention U-Net, with MeanIoU and meanEm improved by up to 1% and 0.07%, respectively. This can show the superiority of the attention mechanism used in the proposed model. Among the models used in the experiments, the proposed model achieves the best results for medical image segmentation.

**Table 4:** The Quantitative result on the SIIM-ACR dataset.

**Dataset**	**Method**	**MeanDice**	**MeanIoU**	**wFm**	**Sm**	**Em**
SIIM-ACR	U-Net (Ronneberger et al., 2015)	0.958	0.922	0.962	0.942	0.977
	Segnet (Badrinarayanan et al., 2017)	0.908	0.835	0.872	0.881	0.937
	R2U-Net (Alom et al., 2018)	0.941	0.891	0.942	0.922	0.968
	Attention U-Net (Oktay et al., 2021)	0.960	0.926	0.920	0.942	0.977
	R2AU-Net (Zuo et al., 2021)	0.859	0.755	0.859	0.851	0.925
	BiSeNet-V2 (Yu et al., 2021)	0.957	0.919	0.961	0.940	0.976
	KiU-Net (Valanarasu et al., 2020)	0.960	0.925	0.963	0.943	0.977
	Transunet (Chen et al., 2021)	0.959	0.922	0.952	0.939	0.975
	GL-Segnet (Ours)	**0.969**	**0.942**	**0.972**	**0.952**	**0.982**

The highest value is marked bold.

#### 4.4.5. Ablation experiment

In order to explore the effects of different factors on model performance, we conducted a series of ablation experiments on the above-mentioned dataset by means of control variables, and this experiment mainly contains the following points:

· The validity of AGs, ViT, and context-rich operations
· The effect of the ViT model's size
· The effect of the location, mode and number of *MSP* and *MSC* modules
· Validity of AGs, ViTs, and context-enrichment operations.

**Validity of AGs, ViT, and context-rich operations:** To further analyze the contributions of AGs, ViT, and context-rich operations in the model, we compared the performance of four variants of the model with U-Net as the backbone, including U-Net, under the above data set by comparing the values of MeanIoU and MeanDice. The experimental results are shown in Table 5, where “U w/AGs+ViT” denotes the U-Net model with the addition of the AGs module and ViT module; “U w/AGs+ER” denotes the U-Net model with the addition of the AGs module and context-rich module; and “U w/ViT+ER” denotes the U-Net model with the addition of the ViT module and the context-rich module.

**Table 5:** Ablation study on the validity of AGs, ViT, and context-rich operations.

**Variants**	**Module**			**Dataset**							
	**AGs**	**ViT**	**ER**	**Glas**		**ISIC**		**Brain Tumors**		**SIIM-ACR**	
				**Mean Dice**	**Mean IoU**	**Mean Dice**	**Mean IoU**	**Mean Dice**	**Mean IoU**	**Mean Dice**	**Mean IoU**
Backbone				0.915	0.847	0.905	0.844	0.922	0.861	0.958	0.922
U w/AGs+ViT	✓	✓		0.929	0.869	0.913	0.854	0.928	0.974	0.966	0.936
U w/AGs+ER	✓		✓	0.924	0.962	0.905	0.846	0.924	0.865	0.967	0.937
U w/ViT+ER		✓	✓	0.927	0.866	0.910	0.850	0.929	0.873	0.965	0.935
GL-Segnet (Ours)	✓	✓	✓	**0.931**	**0.873**	**0.915**	**0.858**	**0.931**	**0.877**	**0.969**	**0.942**

The highest value is marked bold.

From the results in the Table 5, we can see that the MeanDice and MeanIoU of the backbone network on the four datasets are 0.915, 0.847; 0.905, 0.844; 0.922, 0.861; 0.958, 0.922. On each dataset, the indices of the variant model with the addition of the relevant modules are higher than the backbone and lower than the proposed model. The three modules introduced improve the network's ability to extract features to varying degrees, which reflects the effectiveness of the relevant modules introduced in the paper.

**Effect of the ViT model's size:** Two models, “base” and “large”, are experimentally trained. The size of hidden layers, the number of transformer layers and the number of attention headers in each transformer structure are 12, 768, 3,072, and 24, 1,024, 4,096, respectively. The experimental results are shown in Table 6, which shows that with the same number of iterations, the “large” model does not obtain more accurate segmentation results on the GLAS, ISIC, and Brain Tumors datasets and has a huge computing power overhead. Therefore, although its performance on the SIIM-ACR dataset is slightly better than that of the “base” model. we finally chose the “base” model for the experiments.

**Table 6:** Ablation study on the effect of the ViT model's size.

**Variants**	**Dataset**							
	**Glas**		**ISIC**		**Brain Tumors**		**SIIM-ACR**	
	**Mean Dice**	**Mean IoU**	**Mean Dice**	**Mean IoU**	**Mean Dice**	**Mean IoU**	**Mean Dice**	**Mean IoU**
Base	**0.931**	**0.873**	**0.915**	**0.858**	**0.931**	**0.877**	0.969	0.942
Large	0.929	0.871	0.911	0.851	0.929	0.873	**0.971**	**0.945**

The highest value is marked bold.

From the data in Table 6, it can be seen that the “base” model is lower than the “large” model by 0.2 and 0.3 percentage points on the evaluation indexes of Mean Dice and Mean IoU on the SIIM-ACR data, but the two evaluation indexes on the other three data sets are slightly higher than the large model. The “base” model has reached 0.931, 0.915, and 0.931 respectively in the Mean Dice evaluation indicators on the three data sets of Glas, ISIC and Brain Tumors, and is better than the “large” model in terms of model size based on the reason of fewer parameters. Therefore, we finally selected the network structure based on the “base” model.

**Effect of the role position, mode and number of MSC and MSP modules:** To explore the best way to use the *MSC* and *MSP* modules proposed in this paper, four variants of the proposed model with different positions, modes, and numbers of *MSC* and *MSP* modules were trained in this experiment. The experimental results are shown in Table 7, where “L1-SPI” denotes the variant in which the above modules are used serially and act on the jump connection vector of Layer1, and “L2-PRL” denotes the variant in which the above modules are used in parallel and act on the jump connection vector of Layer1. “L2-PRL” denotes the variant model where the above modules are used in parallel and act on the jump connection vector of Layer2. “L3-PRL” denotes the variant model where the above module are used in parallel and act on the jump connection vector of Layer 3, “L123-PRL” denotes the variant model where the above modules are used in parallel and act on the jump connection vector of Layer1, Layer2, Layer3 simultaneously. From the experimental results, we know that the model with the *MSC* and *MSP* modules acting on the jump connection vector of Layer1 in parallel achieves the best experimental results.

**Table 7:** Ablation study on the effect of the role position, mode and number of MSC and MSP modules.

**Variants**	**Dataset**							
	**Glas**		**ISIC**		**Brain Tumors**		**SIIM-ACR**	
	**Mean Dice**	**Mean IoU**	**Mean Dice**	**Mean IoU**	**Mean Dice**	**Mean IoU**	**Mean Dice**	**Mean IoU**
L1-SPI	0.928	0.868	0.906	0.842	0.93	0.875	0.966	0.936
L2-PRL	0.923	0.860	0.910	0.850	0.926	0.868	0.968	0.940
L3-PRL	0.924	0.862	0.91	0.849	0.929	0.873	0.968	0.939
L123-PRL	0.925	0.863	0.912	0.853	0.922	0.860	0.968	0.939
L-PRL (ours)	**0.931**	**0.873**	**0.915**	**0.858**	**0.931**	**0.877**	**0.969**	**0.942**

The highest value is marked bold.

From Table 7, we can clearly see that the model with the L-PRL method is significantly better than the other four methods for the two evaluation metrics on the four data sets. The L-PRL approach outperforms the suboptimal model by 0.5%, 0.5%, 0.2%, and 0.2% on the Mean IoU metric on the four data sets, respectively. Further thinking, the parallel use of *MSC* and *MSP* can maximize the ability of the network to extract features, and the use at the first layer can effectively extract the texture and boundary features of the images.

## 5. Conclusion

In this paper, we attempt to solve the challenge of semantic segmentation of medical images in different medical scenarios, such as image segmentation tasks of rectal adenocarcinoma cells, skin cancerous regions, brain glioma and thoracic organs. In order to cope with the problem of diverse and complex irrelevant background features of medical images in many different medical scenarios, we propose a Global-Local Representation Learning Net for Medical Image Segmentation (GL-Segnet).To solve the intensely interfering irrelevant background information to segment the target, we conceive a Global semantic feature extraction module which can improve the accuracy of model predictions. Nevertheless, to consider simultaneously addressing both long-range and short-range dependencies, and emphasize the geometric detail information implied in the shallow feature maps resulting in the dropping of crucial features, we propose a Context-rich connection module. Experimental results on the four datasets show that the proposed model performs better in medical image segmentation compared to some of the state-of-the-art models. In the future, we will extend the method to segment 3D images and apply this method in an exact medical scenario and modify the model slightly to suit it better.

## Data availability statement

The original contributions presented in the study are included in the article/supplementary material, further inquiries can be directed to the corresponding author.

## Author contributions

Conceptualization and methodology: DG, JZ, YX, and WM. Software: DG, JZ, YX, and PS. Formal analysis: DG, JZ, YX, and HC. Writing—original draft preparation: DG, JZ, YX, HC, ZH, and QW. Writing—review and editing: DG, JZ, YX, ZH, and QW. Supervision: WM. All authors have read and agreed to the published version of the manuscript.

## References

[B1] Ali M. Gu I. Y. H. Berger M. Pallud J. Southwell D. Widhalm G.. (2020). Domain mapping and deep learning from multiple mri clinical datasets for prediction of molecular subtypes in low grade gliomas. Brain Sci. 10, 463. 10.3390/brainsci10070463 32708419 PMC7408150

[B2] Alom M. Z. Hasan M. Yakopcic C. Taha T. M. Asari V. K. (2018). Recurrent residual convolutional neural network based on u-net (r2u-net) for medical image segmentation. J. Med Imaging. 6, 014006. 10.1109/NAECON.2018.8556686 PMC6435980 30944843

[B3] Badrinarayanan V. Kendall A. Cipolla R. (2017). Segnet: a deep convolutional encoder-decoder architecture for image segmentation. IEEE Trans. Pattern Anal. Mach. Intell. 12, 2481–2495. 10.1109/TPAMI.2016.2644615 28060704

[B4] Borovec J. Švihlík J. Kybic J. Habart D. (2017). Supervised and unsupervised segmentation using superpixels, model estimation, and graph cut. J. Electronic Imag. 26, 6. 10.1117/1.JEI.26.6.061610

[B5] Buda M. Saha A. Mazurowski M. (2019). Association of genomic subtypes of lower-grade gliomas with shape features automatically extracted by a deep learning algorithm. Comput. Biol. Med. 109, 218–225. 10.1016/j.compbiomed.2019.05.002 31078126

[B6] Chen J. Lu Y. Yu Q. Luo X. Adeli E. Wang Y.. (2021). TransUNet: Transformers Make Strong Encoders for Medical Image Segmentation

[B7] Cherukuri V. Ssenyonga P. Warf B. C. Kulkarni A.V Monga V. Schiff S. J. (2018). Learning based segmentation of ct brain images: Application to postoperative hydrocephalic scans. IEEE Trans. Biomed. Eng. 65, 871–1884. 10.1109/TBME.2017.2783305 29989926 PMC6062853

[B8] Chu J. Chen Y. Zhou W. Shi H. Cao Y. Tu D.. (2020). “Pay more attention to discontinuity for medical image segmentation,” in Medical Image Computing and Computer Assisted Intervention—*MICCAI 2020*. p. 12264. 10.1007/978-3-030-59719-1_17

[B9] Chu X. Tian Z. Zhang B. Wang X. Shen C. (2021). Conditional positional encodings for vision transformers. arXiv [Preprint]. arXiv: 2006.15320. 10.48550/arXiv.2006.1532036010718

[B10] Deng L. Gong Y. Lin Y. Shuai J. Tu X. Zhang Y.. (2019). Detecting multi-oriented text with corner-based region proposals. Neurocomputing. 334, 134–142. 10.1016/j.neucom.2019.01.013

[B11] Gai D. Shen X. Chen H. Su P. (2020). Multi-focus image fusion method based on two stage of convolutional neural network. Signal Process. 176, 107681. 10.1016/j.sigpro,.2020.10768133525420

[B12] Hua L. Gu Y. Gu X. Xue J. Ni T. (2021). A novel brain MRI image segmentation method using an improved multi-view fuzzy c-means clustering algorithm. Front. Neurosci. 15, 662674. 10.3389/fnins.2021.662674 33841095 PMC8029590

[B13] Huang Z. Wang N. (2017). Like what you like: Knowledge distill via neuron selectivity transfer. arXiv [Preprint]. arXiv: 1707.01219. 10.48550/arXiv.1707.01219

[B14] Jafari M. Auer D. Francis S. Garibaldi J. Chen X. (2020). “Dru-net: An efficient deep convolutional neural network for medical image segmentation,” in Proceedings of the 2020 IEEE 17th International Symposium on Biomedical Imaging. p. 1144–1148.

[B15] Kaiming H. Xiangyu Z. Shaoqing R. (2016). “Deep residual learning for image recognition,” in Proceedings of 2016 IEEE Conference on Computer Vision and Pattern Recognition. p. 770–778.32166560

[B16] Khened M. Kollerathu A. A. Krishnamurthi G. (2018). Fully convolutional multi-scale residual DenseNets for cardiac segmentation and automated cardiac diagnosis using ensemble of classifiers. Med Image Anal. 51, 21–45. 10.1016/j.media.2018.10.004 30390512

[B17] Kitrungrotsakul T. Yutaro I. Lin L. Tong R. Li J. Chen Y. -W. (2020). Interactive deep refinement network for medical image segmentation. arXiv [Preprint]. arXiv: 2006.15320.

[B18] Li H. Zhao X. Su A. Zhang H. Liu J. Gu G. (2018). Color space transformation and multi-class weighted loss for adhesive white blood cell segmentation. IEEE Access. 8, 24808–24818. 10.1109/ACCESS.2020.2970485

[B19] Li Q. Cai W. Wang X. Zhou Y. Feng D. Chen M.. (2014). “Medical image classification with convolutional neural network,” in 13th International Conference on Control Automation Robotics and Vision (ICARCV). p. 844–848. 10.1109/ICARCV.2014.706441435232390

[B20] Li W. Jia F. Hu Q. (2015). Automatic segmentation of liver tumor in ct images with deep convolutional neural networks. J. Comp. Commun. 3, 146–151. 10.4236/jcc.2015.311023

[B21] Li Z. Pan H. Zhu Y. Qin A. K. (2020). “Pgd-unet: A position-guided deformable network for simultaneous segmentation of organs and 2020 tumors, in International Joint Conference on Neural Networks (IJCNN), p. 1–8. 10.1109/IJCNN48605.2020.9206944

[B22] Liu L. Kurgan L. Wu F. Wang J. (2020a). Attention convolutional neural network for accurate segmentation and quantification of lesions in ischemic stroke disease. Med Image Anal. 65, 101791. 10.1016/j.media.2020.101791 32712525

[B23] Liu L. Kurgan L. Wu F. Wang J. (2020b). A survey on u-shaped networks in medical image segmentations. Neurocomputing 244–258. 10.1016/j.neucom.2020.05.070

[B24] Liu X. Wang Z. Li Y. Wang S. (2022). Self-Supervised Learning via Maximum Entropy Coding.

[B25] Liu Z. Lin Y. Cao Y. Hu H. Wei Y. Zhang Z.. (2021). “Swin transformer: Hierarchical vision transformer using shifted windows,” in Proceedings of the IEEE/CVF International Conference on Computer Vision. p. 12100–10022. 10.1109/I.C.C.V.48922.2021.00986

[B26] Mazurowski M. Clark K. Czarnek N. Shamsesfandabadi P. Peters K. Saha A. (2017). Radiogenomics of lower-grade glioma: algorithmically-assessed tumor shape is associated with tumor genomic subtypes and patient outcomes in a multi-institutional study with the cancer genome atlas data. J. Neuro-Oncol. 133, 27–35. 10.1007/s11060-017-2420-1 28470431

[B27] Mortazi A. Bagci U. (2018). “Automatically designing cnn architectures for medical image segmentation,” in International Workshop on Machine Learning in Medical Imaging Springer p. 98–106. 10.1007/978-3-030-00919-9_12

[B28] Oksuz K. Cam B. C. Kalkan S. Akbas E. (2022). One metric to measure them all: Localisation recall precision (lrp) for evaluating visual detection tasks. IEEE Trans. Pattern Anal. Mach. Intell. 44, 844–848. 10.1109/TPAMI.2021.3130188 34813471

[B29] Oktay O. Schlemper J. Le Folgoc L. Lee M. Heinrich M. Misawa K.. (2021). Attention u-net: Learning Where to Look for the Pancreas.35474556

[B30] Petit O. Thome N. Rambour C. Themyr L. Collins T. Soler L. (2021). U-net transformer: Self and cross attention for medical image segmentation. Mach. Learn. Med. Imag. 12, 966. 10.1007/978-3-030-87589-3_2834892009

[B31] Ronneberger O. Fischer P. Brox T. (2015). “U-net: Convolutional networks for biomedical image segmentation,” in Medical Image Computing and Computer-Assisted Intervention-MICCAI 2015. p. 9351. 10.1007/978-3-319-24574-4_28

[B32] San-ping Z. Jin-jun W. Meng-meng Z. Cai Q. Gong Y. (2017). Correntropy-based level set method for medical image segmentation and bias correction. Neuro-computing. 234, 216–229. 10.1016/j.neucom.2017.01.013

[B33] Shankaranarayana S. M. Ram K. Mitra K. Sivaprakash M. (2017). “Joint optic disc and cup segmentation using fully convolutional and adversarial networks,” in Proceedings of International Workshop on Ophthalmic Medical Image Analysis. p. 168–176. 10.1007/978-3-319-67561-9_19

[B34] Sinha A. Dolz J. (2020). “Multi-scale self-guided attention for medical image segmentation,” in IEEE Journal of Biomedical and Health Informatics, Vol. 25 (IEEE), 121–130. 10.1109/JBHI.2020.2986926 32305947

[B35] Song T. Li H. Meng F. Wu Q. Cai J. (2018). Letrist: Locally encoded transform feature histogram for rotation-invariant texture classification. IEEE Trans. Pattern Anal. Mach. Intell. 7, 1565–1579. 10.1109/TCSVT.2017.267189928922134

[B36] Sutton R. T. Pincock D. Baumgart D. Sadowski D. C. Fedorak R. N. Kroeer K. (2020). An overview of clinical decision support systems: benefits, risks, and strategies for success. NPJ Digit. Med. 3, 17. 10.1038/s41746-020-0221-y 32047862 PMC7005290

[B37] Tang B. Chen L. Sun W. Lin Z-k. (2017). Review of surface defect detection based on machine vision. J. Image Graph. 12, 1640–1663. 10.1049/ipr2.12647

[B38] Touvron H. Cord M. Douze M. Massa F. Sablayrolles A. Jegou H. (2021). “Training data-efficient image transformers & distillation through attention,” in Proceedings of the 38th International Conference on Machine Learning (PMLR), 10347-10357. Available online at: https://proceedings.mlr.press/v139/touvron21a.html

[B39] Valanarasu J. Oza P. Hacihaliloglu I. Patel V. (2021). Medical transformer: Gated axial-attention for medical image segmentation. MICCAI. 12901, 36–46. 10.1007/978-3-030-87193-2_436653988

[B40] Valanarasu J. Sindagi V. Hacihaliloglu I. Patel V. (2020). Kiu-net: Towards accurate segmentation of biomedical images using over-complete representations. MICCAI. 12, 264. 10.1007/978-3-030-59719-1_36

[B41] Vaswani A. Shazeer N. Parmar N. Uszkoreit J. Jones L. Gomez A. N.. (2017). “Attention is all you need,” in Proceedings of the 2017 Advances in Neural Information Processing Systems. p. 5998–6008.

[B42] Vorontsov E. Tang A. Pal C. Kadoury S. (2018). “Liver lesion segmentation informed by joint liver segmentation,” in 2018 IEEE 15th International Symposium on Biomedicalimaging (ISBI 2018). p. 1332–1335.35115126

[B43] Yu C. Gao C. Wang J. Yu G. Shen C. Sang N. (2021). Bisenet v2: Bilateral network with guided aggregation for real-time semantic segmentation. Int. J. Comput. Vis. 3051–3068. 10.1007/s11263-021-01515-2

[B44] Zhang Y. Liu H. Hu Q. (2021). “Transfuse: Fusing transformers and cnns for medical image segmentation,” in Medical Image Computing and Computer Assisted Intervention-MICCAI 2021: 24th International Conference (Springer International Publishing). 10.1007/978-3-030-87193-2_2

[B45] Zheng S. Lu J. Zhao H. Zhu X. Luo Z. Wang Y. (2021). “Rethinking semantic segmentation from a sequence-to-sequence perspective with transformers,” in Proceedings of the IEEE/CVF Conference on Computer Vision and Pattern Recognition 6881–6890.

[B46] Zhijie Z. Huazhu F. Hang D. Shen J. Pang Y. Shao L. (2019). “Et-net: A generic edge-attention guidance network for medical image segmentation,” in Proceedings of the 22nd International Conference on Medical Image Computing and ComputerAssisted Intervention. p. 442-450. 10.1007/978-3-030-32239-7_49

[B47] Zhou Z. Rahman Siddiquee M. Tajbakhsh N. Liang J. (2020). Unet++: Redesigning skip connections to exploit multiscale features in image segmentation. IEEE Trans. Biomed. Eng. 6, 1856–1867. 10.1109/TMI.2019.2959609 31841402 PMC7357299

[B48] Zuo Q. Chen S. Wang Z. (2021). “R2AU-Net: attention recurrent residual convolutional neural network for multimodal medical image segmentation,” in Security and Communication Networks. p. 1–10. 10.1155/2021/6625688

